# Fault Reconfiguration in Distribution Networks Based on Improved Discrete Multimodal Multi-Objective Particle Swarm Optimization Algorithm

**DOI:** 10.3390/biomimetics8050431

**Published:** 2023-09-18

**Authors:** Xin Li, Mingyang Li, Moduo Yu, Qinqin Fan

**Affiliations:** 1 Logistics Research Center, Shanghai Maritime University, Shanghai 201306, China; controlxinli@163.com (X.L.); nutmeatss@163.com (M.L.); 2 Key Laboratory of Control of Power Transmission and Conversion, Ministry of Education, Shanghai Jiao Tong University, Shanghai 200240, China; 379440278@sjtu.edu.cn

**Keywords:** distribution network, fault reconfiguration, smart grid, multimodal multi-objective discrete optimization, evolutionary computation

## Abstract

Distribution network reconfiguration involves altering the topology structure of distribution networks by adjusting the switch states, which plays an important role in the smart grid since it can effectively isolate faults, reduce the power loss, and improve the system stability. However, the fault reconfiguration of the distribution network is often regarded as a single-objective or multi-objective optimization problem, and its multimodality is often ignored in existing studies. Therefore, the obtained solutions may be unsuitable or infeasible when the environment changes. To improve the availability and robustness of the solutions, an improved discrete multimodal multi-objective particle swarm optimization (IDMMPSO) algorithm is proposed to solve the fault reconfiguration problem of the distribution network. To demonstrate the performance of the proposed IDMMPSO algorithm, the IEEE33-bus distribution system is used in the experiment. Moreover, the proposed algorithm is compared with other competitors. Experimental results show that the proposed algorithm can provide different equivalent solutions for decision-makers in solving the fault reconfiguration problem of the distribution network.

## 1. Introduction

With the rapid development of industry and technology, the complexity of the distribution network is higher due to access to distributed energy sources and other factors [1]. Moreover, the fault rate of the distribution network is increasing [1], which has a direct impact on the safety of the power supply and satisfaction of customers. Additionally, this can have negative effects on the economic interests of power supply enterprises. An effective method is to reconfigure the distribution network [2] via adjusting the network’s topology when faults occur. Generally, the reconfiguration of distribution networks can be classified into two distinct categories [3]: (1) reconfigure the distribution networks during normal conditions to enhance economic efficiency; (2) fault reconfiguration. The fault reconfiguration is to change the topology structure of the distribution network by adjusting switch states when faults occur. The objective [4] of the fault reconfiguration in the distribution network is to enhance the self-healing capability. Therefore, the fault reconfiguration in the distribution network is a crucial approach for improving the reliability [5] and optimizing the distribution of the power flow [6]. In addition, it is a complex and challenging optimization problem. However, most existing studies regarded such problems as a single-objective or multi-objective optimization problem to solve. For example, to effectively solve the distribution network reconfiguration (DNR) problem, Wang et al. [7] proposed a parallel slime mold algorithm (PSMA), in which the multi-objective DNR model is transformed into a single objective problem via the analytic hierarchy process (AHP) method. The experimental results demonstrate that the PSMA is an effective approach to address DNR problems. Badran et al. [8] proposed a simultaneous optimization approach to effectively address the network reconfiguration problem. The experimental results demonstrate that the proposed approach performs well in realizing optimal network configuration and distributed generation output. Yang et al. [9] developed a fault recovery reconstruction model for the distribution network, whose objective is to minimize the network loss. Then, a differential evolution algorithm with enhanced coding and mutation strategies is proposed to solve the above proposed problem. The effectiveness of the proposed method is validated through the IEEE33-bus distribution system. Mahdavi et al. [10] proposed a reconfiguration model with the objective of mitigating the power loss of the distribution network. Moreover, an innovative computational technique is proposed to improve power efficiency and voltage characteristics. Additionally, the proposed technique is combined with the whale optimization algorithm to solve the model. The experimental results demonstrate that the proposed approach is effective in solving the reconfiguration problems of the distribution network. Different from the above studies, Eldurssi et al. [11] proposed an improved fast nondominated sorting genetic algorithm to solve the reconfiguration problem of the distribution system. Moreover, a novel mutation operator and a verification method of the system radiality are presented. The experimental results demonstrate that the proposed approach is competitive. Zhong et al. [12] proposed a multi-objective reconfiguration problem of the distribution network. Then, an improved multi-objective Bayesian learning-based evolutionary algorithm is utilized to solve the proposed problem. The experimental results demonstrate that the proposed algorithm can efficiently converge using a small population. Nguyen et al. [13] proposed a runner root algorithm (RRA) to address the DNR problem. In the RRA, random jumps and re-initialization strategies are incorporated to prevent trapping into local optima. The experimental results show that the RRA has an excellent performance in solving the DNR problem. Li et al. [14] proposed an integrated optimization method for addressing the dynamic reconstruction of an active distribution network (ADN). Moreover, a multi-objective sparrow search algorithm (MOSSA) is proposed to solve the above problem. The experimental results demonstrate that the MOSSA can enhance the stability of the ADN. To enhance the operational efficiency of the distribution network, Qi et al. [15] proposed a multi-objective optimization problem to reduce the power loss, balance the load, and improve the voltage profile. Moreover, an innovative optimization approach, in which a local search method is combined with a multi-objective particle swarm optimization algorithm, is introduced to achieve the above objectives. The experimental results indicate that the proposed method is effective. Evidently, the reconfiguration problem in the distribution network is considered as a single-objective or multi-objective optimization problem in the above-mentioned studies; thus, its multimodality is neglected. Therefore, the obtained solutions may be infeasible or unsuitable when the environment changes.

In the real world, many multi-objective optimization problems (MOPs) have equivalent solutions, which are called multi-modal MOPs (MMOPs). To effectively solve MMOPs, Fan et al. [16] proposed a zoning search (ZS) method to reduce the search difficulty to find more equivalent solutions. Subsequently, Fan et al. [17] proposed the ZS with adaptive resource allocating (ZS-ARA) method to enhance the search efficiency of the ZS method. To find more equivalent solutions for multi-robot task allocation (MRTA) problems, Miao et al. [18] proposed an improved multimodal multi-objective differential evolutionary algorithm hybrid with a simulated annealing algorithm. Clearly, MMOPs can be found in different fields. For the fault reconfiguration of the distribution network, there may be multiple equivalent schemes corresponding to the same objective in some cases. Therefore, finding these equivalent schemes is an important and challenging task to enhance the reliability and safety of the distribution network. To carry out the above objectives, an improved discrete multimodal multi-objective particle swarm optimization (IDMMPSO) algorithm is proposed in the current study. In the IDMMPSO algorithm, a novel decision space crowding distance calculation method based on the Hamming distance is utilized to compute the distances among individuals. Moreover, an enhanced environment selection method is used to improve the population diversity in the decision space. To demonstrate the performance of the proposed algorithm, three famous multi-objective evolutionary algorithms (i.e., NSGA-II [19], SPEA2 [20], and MOPSO [21]) are selected in experiments. Additionally, the IEEE33-bus distribution system [22] is utilized to test the performances of all competitors.

The main contributions of this paper are follows:

(1) Different from previous studies, the current study considers not only the multi-objective in the fault reconfiguration of the distribution network but also its multimodality. Therefore, more equivalent schemes can be provided in the proposed algorithm, which can help decision-makers address the fault reconfiguration problem of distribution networks in uncertain/dynamic environments.
(2) Although various multimodal multi-objective evolutionary algorithms have been proposed in existing studies, most of them are not applicable to solve discrete optimization problems. To alleviate this issue, an improved multimodal multi-objective particle swarm algorithm is proposed in the current study. In the proposed algorithm, the Hamming distance is employed to evaluate the similarity of discrete vectors in the decision space.

## 2. Fault Reconfiguration Model of Distribution Network

The fault reconfiguration in the distribution network is a complex and large-scale nonlinear programming problem. Moreover, to reduce the power loss and the voltage deviation, a novel fault reconfiguration problem of the distribution network is proposed in the present study. More details are given as follows.

### 2.1. Objective Function

Based on Refs. [22,23,24,25], power loss and voltage deviation are commonly used as objective functions in the reconfiguration of the distribution network. These two objectives can help to reduce the system losses and maintain the network stability. Therefore, the power loss and the voltage deviation are two objective functions in the present study.

#### 2.1.1. Power Loss

The formula of the power loss can be described as follows:

(1) $$f_{1}=\min\sum_{ij=1}^{M}k_{ij}R_{ij}\frac{P_{ij}^{2}+Q_{ij}^{2}}{U_{j}^{2}},$$

where *M* represents the number of branches; *k_ij_* denotes the switching state of branch *ij*; *R_ij_* refers to the impedance of branch *ij*; *P_ij_* and *Q_ij_* represent the active and reactive power flowing through branch *ij*, respectively; and *U_ij_* is the terminal voltage of branch *ij* and is the actual voltage of node *j*.

#### 2.1.2. Voltage Deviation

The formula of the voltage deviation can be expressed as follows:

(2) $$f_{2}=\min\sum_{j=1}^{N}\frac{\left|U_{j}-U_{j,a}\right|}{U_{j,a}},$$

where *N* refers to the number of nodes; *U_j_* is the actual voltage of node *j*; and *U*_*j*,*a*_ is the rated voltage of node *j*.

### 2.2. Constraints

#### 2.2.1. Power Balance Constraint

Mathematically, the power balance constraint can be defined as follows:

(3) $$\left\{\begin{array}{l} P_{i}-U_{i}\sum_{j\in i}U_{j}\left(G_{ij}\cos\theta_{ij}+B_{ij}\sin\theta_{ij}\right)=0\\ Q_{i}-U_{i}\sum_{j\in i}U_{j}\left(G_{ij}\sin\theta_{ij}+B_{ij}\cos\theta_{ij}\right)=0 \end{array}\right.,$$

where *P_i_* and *Q_i_* are the active and reactive power injected into node *i*, respectively; *G_ij_*, *B_ij_*, and *θ_ij_* are the conductance, electrical susceptance, and phase angle difference between node *i* and node *j*, respectively.

#### 2.2.2. Node Voltage Constraint

The limitation of the node voltage can be described as follows:

(4) $$U_{i}^{\min}\leq U_{i}\leq U_{i}^{\max},$$

where $U_{i}^{\min}$ and $U_{i}^{\max}$ are the lower and upper voltage limits at node *i*, respectively.

#### 2.2.3. Branch Current Constraint

The branch current constraint can be expressed as follows:

(5) $$I_{ij}\leq I_{ij}^{\max},$$

where $I_{ij}$ is the actual current of branch *ij* and $I_{ij}^{\max}$ is the upper limit of $I_{ij}$.

#### 2.2.4. Topology Constraint

The radial topology of the distribution network can be described as follows:

(6) $$h_{k}\in H_{k},$$

where *h_k_* is the reconfigured network topology, and *H_k_* is the network topology that conforms to the operation rules of the distribution network.

## 3. The Proposed Algorithm

Because the fault reconfiguration problem of the distribution network is a discrete multimodal multi-objective optimization problem (DMMOP), a novel discrete multimodal multi-objective particle swarm optimization algorithm is proposed in the current study. In the proposed algorithm, the binary PSO [26] is used as the search engine. Moreover, the ring topology strategy [27] is utilized to maintain the population diversity. Additionally, an improved crowding distance calculation method, which is well suited for discrete space, is proposed to assess the crowding degree between individuals.

### 3.1. Encoding Method

Because the topology structure of the distribution network can be changed by closing or opening switches, switches are used as optimization variables. Clearly, the fault reconfiguration of the distribution network is a discrete optimization problem, in which the variables are the states of switches. Therefore, in the proposed algorithm, “0” represents an open switch state, while “1” represents a closed switch state.

### 3.2. Crowding Distance in the Decision Space Based on Hamming Distance

Since the fault reconfiguration of the distribution network is a discrete optimization problem, it is difficult to measure the crowding degree of individuals in the decision space. To solve the above issue, the Hamming distance method [28] is utilized to measure the similarity of two discrete vectors in the decision space. For each individual in the population, its decision space crowding distance is calculated as follows:

Step 1: Randomly select an individual *x_j_* from the population and then calculate the distances between *x_j_* and remaining individuals in this population via the Hamming distance method.

Step 2: Designate *x_j_* as the first individual and sort the remaining individuals in this population according to their Hamming distances via Step 1. If there are individuals with the same distance, they will be randomly sorted from front to back.

Step 3: Set the decision space crowding distance of the first individual *x_j_* to 0, denoted as *CD*_*j*,*x*_ (*CD*_1*,x*_); and the decision space crowding distance of the last individual to 1, denoted as *CD*_*n*,*x*_. The crowding distance of the remaining individuals is calculated as follows:

(7) $$CD_{k,x}=\left\{\begin{array}{l} 0\;\;\;\;\;\;\;\;\;,\;\mathrm{if}\;k=1\\ \frac{\left(x_{k-1}⊕x_{k})+(x_{k}⊕x_{k+1}\right)}{HM_{\max}},\;\mathrm{if}\;1<\;k<n\\ 1\;\;\;\;\;\;\;\;\;,\;\mathrm{if}\;k=n \end{array}\right.,$$

where $CD_{k,x}$ is the decision space crowding distance of individual $x_{k}$; $x_{k-1}⊕x_{k}$ refers to the Hamming distance between $x_{k-1}$ and $x_{k}$; $HM_{\max}$ refers to the maximum Hamming distance in Step 1; and *n* is the size of this population.

### 3.3. Environment Selection Method

The distribution network system needs to comply with a radial topology [22]; thus, the graph theory [29] and a branch-exchange method [22] are employed to describe its structure and handle the topology constraint, respectively. Moreover, the conventional power flow calculation method [30] is utilized to calculate the voltage and current values for each node in the distribution network system. Furthermore, the obtained results may be infeasible. To solve this issue, the penalty method [31] is used in the proposed algorithm. In other words, if the constraints are violated, a large penalty will be given.

Besides the above issues, one of the challenges in the multimodal multi-objective optimization is to preserve individuals with a small objective space crowding distance but a large decision space crowding distance during the environment selection [32]. Different from general MOPs, MMOPs aim to find more equivalent solutions in the decision space. Based on Ref. [33], the non-dominated_scd_sort method is utilized to balance the population diversity of the objective space and decision space in the current study. Note that the original non-dominated_scd_sort method is not applicable to solve discrete optimization problems. Therefore, according to Section 3.2, the Hamming distance is utilized to calculate the crowding distance in the non-dominated_scd_sort method, which is named as the INSCD.

### 3.4. Overall Implementation of IDMMPSO Algorithm

To effectively solve the fault reconfiguration problem of the distribution network, the IDMMPSO algorithm is proposed in the current study. It should be noted that some operators in the IDMMPSO algorithm are similar as those in the MO_Ring_PSO_SCD [33], such as the search engine (i.e., PSO) and environmental selection method (i.e., non-dominated_scd_sort method). The velocity and position of the binary particle are updated as follows:

(8) $$v_{i,d}^{t+1}=\omega \cdot v_{i,d}^{t}+c_{1}r_{1}(pbest_{i,d}^{t}-x_{i,d}^{t})+c_{2}r_{2}(nbest_{i,d}^{t}-x_{i,d}^{t}),$$

(9) $$x_{i,d}^{t+1}=\left\{\begin{array}{l} 1\;,\;if\;rand\;<\;\mathrm{logsig}(v_{i,d}^{t+1})\\ 0\;,\;if\;rand\geq \mathrm{logsig}(v_{i,d}^{t+1}) \end{array}\right.,$$

where $v_{i,d}^{t}$ and $x_{i,d}^{t}$ denote the velocity and position of the *d*-th dimension of the *i*-th particle at the *t*-th generation, respectively; *ω* is the inertia factor; *c*_1_ and *c*_2_ are the learning factors; *r*_1_ and *r*_2_ are two random values independently in the interval [0, 1]; *pbest* and *nbest* are the positions of historical and neighborhood optimal particle, respectively; logsig is a commonly used activation function that maps input values to outputs between 0 and 1. The pseudocode of the IDMMPSO is shown in Algorithm 1.

An initial population is generated in line 1, and the historical optimal archive ***HOA*** and the neighbor optimal archive ***NOA*** are initialized in line 2. ***HOA****{i}* is used to store the historical optimal positions of the *i*-th individual, while ***NOA****{i}* is employed to save the optimal positions within the neighbors of the *i*-th individual. Line 4 is used to judge the termination condition of the IDMMPSO algorithm. Line 6 is to sort all particles in ***HOA*** and ***NOA*** via the INSCD method. In line 7, *pbest_i_* and *nbest_i_* are selected from ***HOA****{i}* and ***NOA****{i}*, respectively. Line 8 updates ***P****_i_*(*t*) to ***P****_i_*(*t* + 1) via Equations (8) and (9). Subsequently, the environmental selection method (see Section 3.3) is used to select individuals. Specifically, the conventional power flow calculation method [30] is utilized to calculate the voltage and current values for each node of ***P****_i_*(*t* + 1) in line 9. Moreover, the penalty method [31] is utilized to handle the infeasible individual in lines 10–12. Line 13 is to save ***P****_i_*(*t* + 1) in ***HOA****{i}*. The INSCD method is utilized to update the ***HOA*** *{i}* in line 14. In lines 16–18, select non-dominated individuals from ***HOA*** *{i − 1}*, ***HOA*** *{i}*, and ***HOA*** *{i + 1}* then store them in ***NOA****{i}* (i.e., the ring topology strategy). Finally, output all non-dominated individuals in ***NOA***, i.e., the states of switches in the distribution network.
**Algorithm** **1:** Framework of IDMMPSO.**Input:** the size of population: *NP*; the dimension of particle: *D*; maximum number of iterations: *T*;1:Initialize the population ***P***(0);2:Initialize the historical optimal archive ***HOA*** and neighbor optimal archive ***NOA***;3:*t* = 1;4:**while** *t < T* **do**5:   **for** *i* = 1: *NP* **do**6:      Sort all particles in ***HOA*** and ***NOA*** via the INSCD method;7:      *pbest_i_* and *nbest_i_* are selected from the first particle in ***HOA****{i}* and ***NOA****{i}*, respectively;8:      Update ***P****_i_*(*t*) to ***P****_i_*(*t* + 1) via Equations (8) and (9);9:      The voltage and current values for each node of ***P****_i_*(*t* + 1) are calculated by the power flow calculation method;10:      **if *P****_i_*(*t* + 1) violates constraints **then**11:         Give ***P****_i_*(*t* + 1) a large penalty via the penalty method;12:      **end**13:      Save ***P****_i_*(*t* + 1) in ***HOA****{i}*;14:      Update ***HOA****{i}* via the INSCD method;15:     **end for**16:     **for** *i* = 1: *NP* **do**17:      Select non-dominated individuals from ***HOA****{i − 1}*, ***HOA****{i}*, and ***HOA****{i + 1}* and save them in ***NOA****{i}*;18:     **end for**19:     *t* = *t* + 1;20:**end while****Output:** All the non-dominated individuals in ***NOA***.

## 4. Experimental Comparisons and Analysis

The performance of the IDMMPSO algorithm is compared with other state-of-the-art multi-objective algorithms on the IEEE 33-bus distribution system [22]. Five performance metrics are utilized to assess the performance of all competitors, which are the hypervolume metric (HV) [34], the binary coverage metric (C) [35], the spacing metric (SP) [36], the inverted generational distance based on the synthetic optimal Pareto front (IGD-CF) [37], and an improved PSP-D metric based on the Pareto set proximity (PSP) [33] and Hamming distance [28]. Similar to the HV, a larger C value and PSP-D value means the performance of the algorithm is better. Conversely, a smaller SP value and IGD-CF value denote that the performance of the algorithm is superior.

### 4.1. Parameter Settings

**Figure 1:**
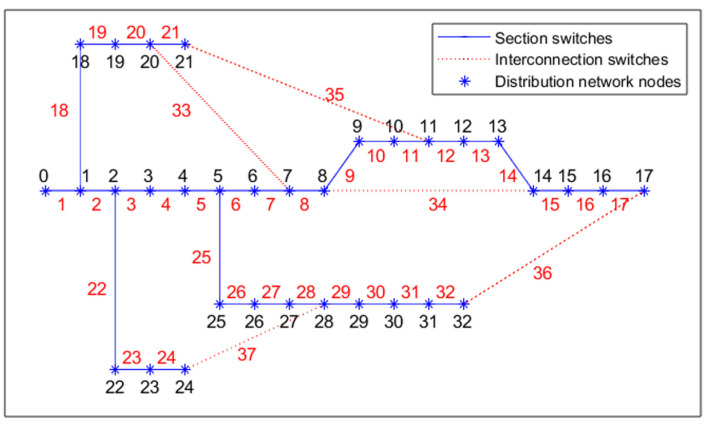
IEEE 33-bus distribution system.

**Figure 2:**
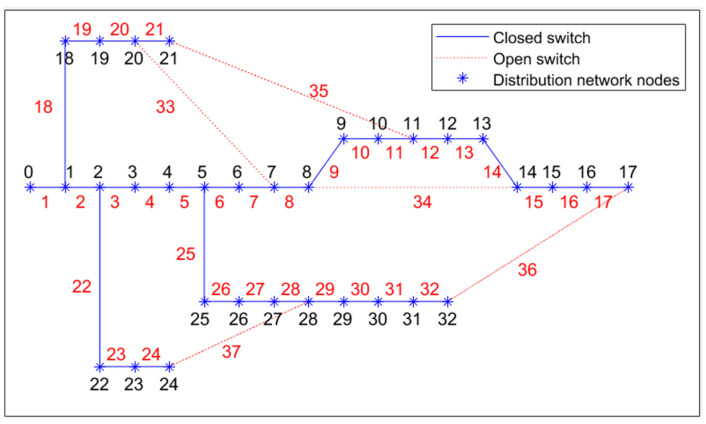
Initial state topology of the distribution network.

In order to verify the effectiveness and superiority of the IDMMPSO algorithm, the IEEE 33-bus distribution system [22] shown in Figure 1 is used in the experiments, which contains 33 nodes, 32 section switches, and 5 interconnection switches. Moreover, the IDMMPSO algorithm and the other three algorithms are implemented with MATLAB 2021a. The base voltage of the IEEE 33-bus distribution system is 12.66 kV. The initial state of the distribution network before a fault occurs is shown in Figure 2. It can be observed from Figure 2 that the switches 33, 34, 35, 36, and 37 are opened, and the remaining switches are closed. Moreover, the initial power loss and voltage deviation are 191 kW and 0 p.u., respectively. More details can be found in [22]. The parameters of the search engine PSO are the same as the original Ref. [26]. For all the compared algorithms, the maximum numbers of function evaluations and population size are set to 10,000 and 100, respectively.

### 4.2. Comparison with Other Competitors

To verify the superiority of the IDMMPSO algorithm, it is assumed that branch 9 faults occur. The performance of the IDMMPSO algorithm is compared with that of three other competitive discrete multi-objective optimization algorithms, namely the NSGA-II [19], the MOPSO [21], and the SPEA2 [20]. The experiment is conducted with each algorithm running 21 times, and the best results are highlighted in bold. The Wilcoxon non-parametric test [38] is utilized to analyze the experimental data. The symbols “+”, “−“, and “≈” indicate whether the IDMMPSO algorithm outperformed, underperformed, or was comparable with the compared algorithms.

Table 1 presents the average and standard deviation values of five performance metrics obtained by four comparison algorithms. From Table 1, it is observable that the IDMMPSO algorithm outperforms the NSGA-II, MOPSO, and SPEA2 in terms of IGD-CF, HV, C, and SP. This means that the IDMMPSO algorithm can find a high-quality Pareto front approximation in the objective space in solving the fault reconfiguration problem of the distribution network. Moreover, it can be observed that the IDMMPSO algorithm outperforms the NSGA-II, MOPSO, and SPEA2 in terms of PSP-D. This indicates that the IDMMPSO algorithm can obtain more high-quality equivalent solutions in the decision space. The main reason may be that the used search engine and the proposed environmental selection method can help the proposed IDMMPSO algorithm to find high-quality solutions and maintain the population diversity. Therefore, it can be concluded that the proposed IDMMPSO algorithm is a competitive method to solve the fault reconfiguration problem of the distribution network and can help decision-makers address various complex or uncertain scenarios.

### 4.3. Multimodality of Solutions

As stated above, finding equivalent schemes is important in solving the fault reconfiguration problem of the distribution network. Therefore, the performance of the IDMMPSO algorithm is further demonstrated using the IEEE 33-bus distribution system in the current experiment.

**Figure 3:**
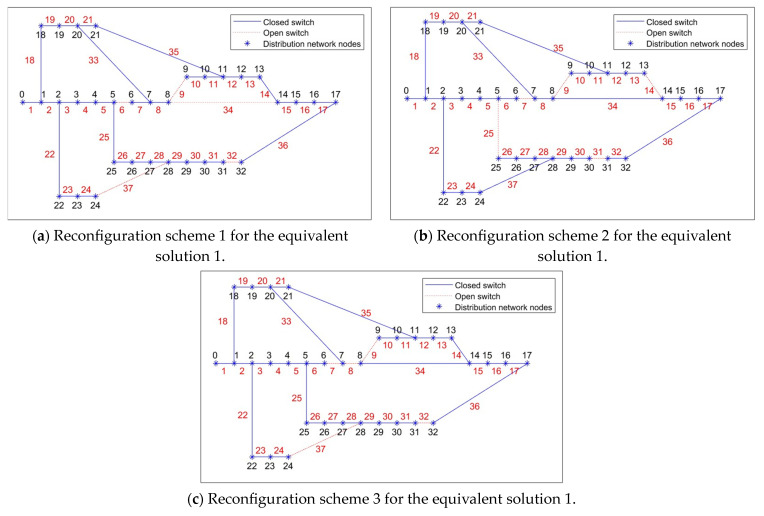
Reconfiguration schemes of equivalent solution 1 obtained by the proposed problem.

**Figure 4:**
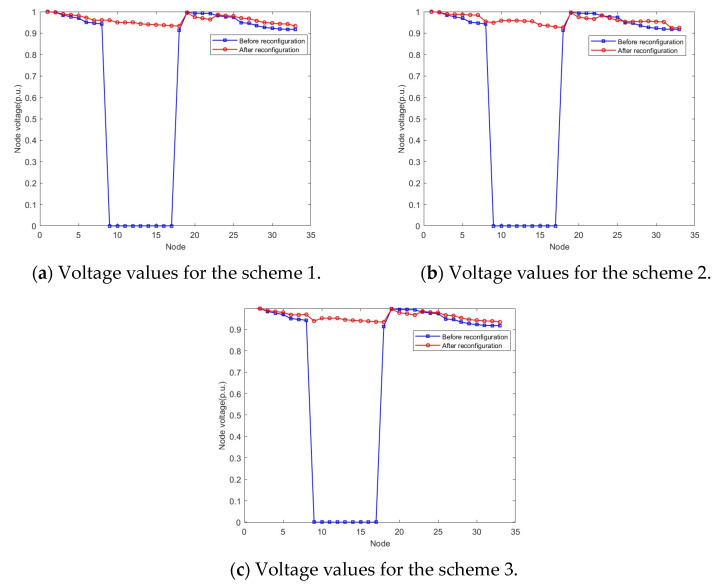
Voltage values of each node for the equivalent solution 1.

**Figure 5:**
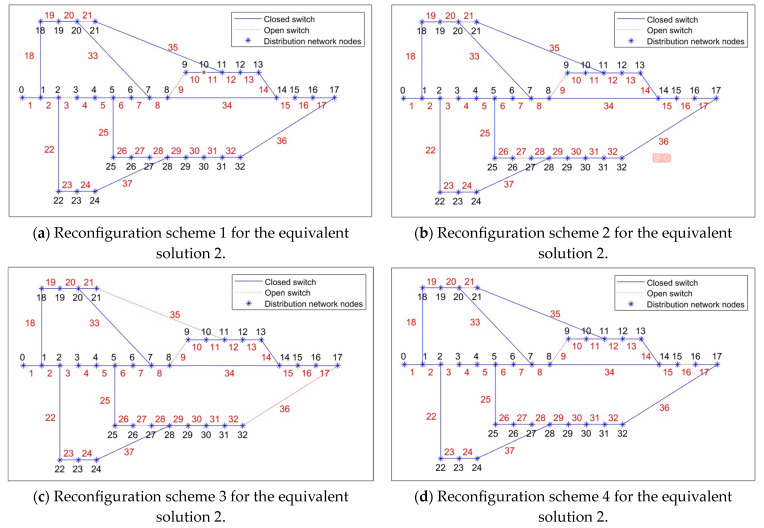
Reconfiguration schemes of equivalent solution 2 obtained by the proposed problem.

**Figure 6:**
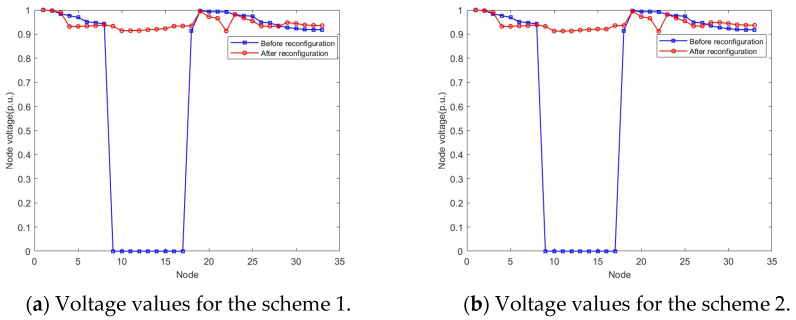

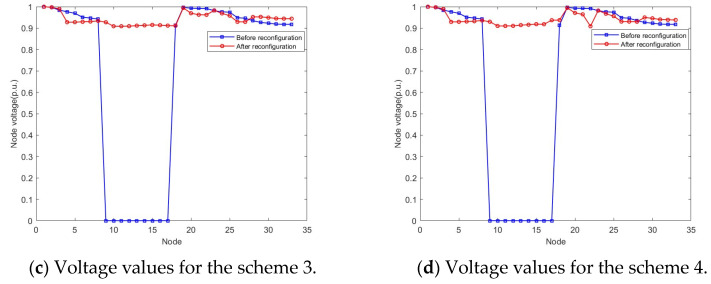
Voltage values of each node for the equivalent solution 2.

Two typical equivalent solutions are shown in Table 2. It can be observed from Table 2 that, for the “equivalent solution 1”, their objective function values (i.e., the power loss and the voltage deviation) in the objective space are similar while the Pareto optimal solutions in the decision space are different. Three equivalent schemes are illustrated in Figure 3. It can be observed from Figure 3 that, if the switch “6” in the scheme “6–9–32–34–37” cannot be used, then two equivalent schemes “7–9–14–25–31” and “7–8–9–32–37” can achieve the same objective. Furthermore, the node voltage values before and after reconfiguration of the three schemes are shown in Figure 4. Because nodes 9 to 17 are de-energized due to the fault in branch 9, the voltage values of these nodes are equal to 0 before reconfiguration. However, Figure 4 shows that all de-energized nodes are restored, the minimum node voltage has increased, and voltage quality of the power supply has improved after the distribution network is reconfigured in all three schemes. Clearly, the proposed algorithm is capable of adapting dynamic/uncertain environments. For the “equivalent solution 2”, we can achieve the same conclusion to the “equivalent solution 1”, i.e., the obtained equivalent solutions can assist decision-makers in addressing unexpected faults, thus completing the fault reconfiguration of the distribution network without adjusting the objectives. The equivalent schemes of the “equivalent solution 2” are illustrated in Figure 5. Additionally, the node voltage values of these schemes before and after reconfiguration are illustrated in Figure 6. It can be observed that all de-energized nodes are restored and the node voltages of the 33 nodes in the four schemes are within the range of [0.9, 1.1] p.u. [22,39]. Therefore, the above four schemes are feasible after reconfiguring the distribution network.

Based on the above analyses, we can conclude that the proposed algorithm can provide equivalent schemes to help decision-makers to address the fault reconfiguration problem of the distribution network in complex or uncertain environments. This can significantly enhance the safety and reliable of the distribution network.

### 4.4. Computational Time Analysis

The execution time and performance of an algorithm are two crucial performance indicators for solving actual problems. Therefore, the execution time and the obtained solutions of all compared algorithms are further investigated in this section. The parameter settings are the same as those in Section 4.1. The experiment is conducted with each algorithm running 21 times.

For all competitors, the average execution time and the number of obtained solutions are presented in Table 3. It can be observed from Table 3 that the computational time of the proposed algorithm is shorter than that of the MOPSO and SPEA2. Moreover, the IDMMPSO algorithm can obtain more solutions when compared with the MOPSO and SPEA2. Table 3 also indicates that, although the run time of the NSGA-II is shorter than that of the proposed algorithm, the NSGA-II cannot find more solutions when compared with the IDMMPSO algorithm. Additionally, the computational time of the proposed algorithm is acceptable. Therefore, the IDMMPSO algorithm is an effective and available tool for solving the fault reconfiguration problem in the distribution network.

## 5. Conclusions

The fault reconfiguration of the distribution network is vital for enhancing the efficiency and reliability of the smart grid. In this paper, an improved discrete multimodal multi-objective particle swarm optimization (IDMMPSO) algorithm is proposed to solve the fault reconfiguration problem of the distribution network. In the IDMMPSO algorithm, an improved environment selection method is proposed to solve DMMOPs. To demonstrate the effectiveness of the IDMMPSO algorithm, it is compared with MOPSO, NSGA2 and SPEA2 algorithms on the IEEE33-bus distribution system. The results demonstrate that the IDMMPSO algorithm is a competitive tool to solve the fault reconfiguration problem of the distribution network. Moreover, the IDMMPSO algorithm can obtain more equivalent solutions for decision-makers to deal with emergencies and changing environments.

## Figures and Tables

**Table 1:** Results of all comparison algorithms in terms of IGD-CF, HV, C, SP, and PSP-D.

Metrics	NSGA-II		MOPSO		SPEA2		IDMMPSO
IGD-CF	7.11 × 10^−3^ (1.56 × 10^−3^)	+	5.33 × 10^−3^ (9.73 × 10^−4^)	+	8.92 × 10^−3^ (1.65 × 10^−3^)	+	**3.98 × 10^−3^ (8.22 × 10^−4^)**
HV	6.01 × 10 ^0^ (7.21 × 10^−2^)	+	6.04 × 10 ^0^ (4.82 × 10^−2^)	+	6.02 × 10 ^0^ (7.34 × 10^−2^)	+	**6.06 × 10 ^0^ (2.75 × 10^−2^)**
C	9.38 × 10^−1^ (5.98 × 10^−2^)	+	9.71 × 10^−1^ (5.03 × 10^−2^)	+	9.54 × 10^−1^ (5.74 × 10^−2^)	+	**9.84 × 10^−1^ (3.09 × 10^−2^)**
SP	2.28 × 10^−2^ (3.82 × 10^−3^)	+	1.95 × 10^−2^ (2.58 × 10^−3^)	+	2.12 × 10^−2^ (3.57 × 10^−3^)	+	**1.77 × 10^−2^ (3.38 × 10^−3^)**
PSP-D	5.99 × 10 ^2^ (5.20 × 10 ^1^)	+	7.52 × 10 ^2^ (4.89 × 10 ^1^)	+	8.13 × 10 ^2^ (5.00 × 10 ^1^)	+	**1.14 × 10 ^3^ (1.37 × 10 ^2^)**
+		5		5		5	
−		0		0		0	
≈		0		0		0	

**Table 2:** The equivalent solutions obtained by the proposed algorithm.

	Solutions					Power Loss/100 kW	Voltage Deviation/p.u.
Equivalent solution 1	6	9	32	34	37	1.36	1.72
	7	9	14	25	31	1.37	1.71
	7	8	9	32	37	1.38	1.71
Equivalent solution 2	3	9	15	21	28	1.91	1.07
	3	9	16	21	27	1.91	1.08
	3	9	27	35	36	1.89	1.08
	3	9	16	21	28	1.92	1.06

**Table 3:** The average execution time and the number of solutions of all algorithms.

	NSGA-II	MOPSO	SPEA2	IDMMPSO
**The execution time (s)**	35.27	64.32	63.91	59.84
**The number of solutions**	36	39	40	51

## Data Availability

Data supporting reported results are available from the authors upon reasonable request.
